# Prevalence of Coexistent Plantaris Tendon Pathology in Patients with Mid-Portion Achilles Pathology: A Retrospective MRI Study

**DOI:** 10.3390/sports7050124

**Published:** 2019-05-22

**Authors:** Sachin Khullar, Prasanna Gamage, Peter Malliaras, Leesa Huguenin, Ashutosh Prakash, David Connell

**Affiliations:** 1 Australasian College of Sports and Exercise Medicine, Melbourne 3000, Australia; leesakym@gmail.com; 2 MP Sports Physicians, Victoria 3199, Australia; 3 Australian Centre for Research into Injury in Sport and its Prevention (ACRISP), School of Medical and Health Sciences, Edith Cowan University, Joondalup 6027, Australia; jpgamage@yahoo.com; 4 Department of Physiotherapy, Monash University, Melbourne 3199, Australia; peter.malliaras@monash.edu; 5 Imaging @ Olympic Park, Melbourne 3004, Australia; drashutoshprakash@gmail.com (A.P.); davidconnellrad@googlemail.com (D.C.); 6 Tan Tock Seng Hospital, Singapore 308433, Singapore; 7 Department of Radiology, Monash University, Melbourne 3800, Australia

**Keywords:** Achilles, mid-portion, tendinopathy, Plantaris

## Abstract

Co-existence of Plantaris tendinopathy (PT) in patients with mid-Achilles tendinopathy (Mid-AT) is of clinical significance. This study aims to describe the MRI-based pathological characteristics of co-existing PT and Mid-AT. One-hundred MRI studies of patients diagnosed with Mid-AT were retrospectively analysed by an experienced musculoskeletal radiologist. Presence or absence of a Plantaris tendon, co-existing PT with Mid-AT, insertional characteristics of Plantaris tendon, and maximum anteroposterior thickness of the tendon in Mid-AT (axial images) were evaluated. When PT co-existed with Mid-AT, the location of the tendon pathologies in relation to calcaneal insertion was assessed (sagittal images) and their association was analysed using the coefficient of variation (CV) and Pearson’s correlation coefficient. Plantaris was present in 84 cases (84%), and Mid-AT and PT co-existed in 10 cases (10%). A greater variability in the location of Plantaris pathology (CV = 42%) than Achilles tendinopathy (CV = 42%) was observed. The correlation coefficient also revealed a low and non-significant association between the location of two pathologies when they exist together (r = +0.06; *p* = 0.88). Clinical evaluation of Achilles tendon pain needs careful consideration into the possible co-existence of Plantaris pathology. The considerable difference observed in the location of PT and Mid-AT suggest possible isolated pathologies and differentials for Achilles tendon pain.

## 1. Introduction

Plantaris tendon pathology is a potential differential diagnosis for Achilles tendinopathy [1]. The Plantaris muscle originates at the lower part of the lateral femoral supracondylar line and the oblique popliteal ligament [2,3]. Its small fusiform belly is 7–10 cm long and ends in a long slender tendon, which crosses obliquely in an inferomedial direction between gastrocnemius and soleus muscles [2,3]. The tendon runs distally along the medial border of the Achilles tendon and typically inserts onto the calcaneus just medial to the Achilles tendon [2,3]. However, a considerable anatomical variability has been observed in Plantaris muscle, especially in relation to its insertion [4,5]. The Plantaris muscle is considered to be a vestigial remnant, and several studies have reported it to be absent in some individuals [2,3,6].

Achilles tendinopathy is a common cause of lower limb pain characterised by localised tendon pain, tendon pathology and dysfunction [7]. Amongst professional runners, 42% experience Achilles tendinopathy before the age of 45 years, compared to 10% in the general population [8]. Although Achilles tendinopathy is common among runners, it is estimated that up to 65% of cases in the general population may not involve sports activities [9]. The pathophysiology of Achilles tendinopathy is complex and is thought to involve a mismatch between load demands on the tendon and adaptive potential that results in altered cell signalling and tissue pathology [10]. A number of risk factors have been identified for Achilles tendinopathy, categorised into extrinsic factors including activity and load on the tendon and intrinsic factors such as age, genetic profile, and metabolic factors (e.g., elevated cholesterol or diabetes) [7,10,11].

In recent years, there has been increased interest in the presence of Plantaris tendon and its anatomical variations associated with developing Achilles tendinopathy [12]. Further, co-existence of Plantaris tendinopathy (PT) with mid-portion Achilles tendinopathy (Mid-AT) have been reported. This may have significant clinical importance in the diagnosis and management of Mid-AT [4,13,14,15]. Several studies have focused on the histo-pathological and cadaveric appearance of the Plantaris tendon in relation to Achilles tendinopathy [7,16,17]. Spang et al., [1] reported that the Plantaris tendon lies invaginated or very close to the Achilles tendon, and showed degenerative features similar to those in co-existing Achilles tendinopathy. They also noted an increase in local Acetylcholine production, which is a marker of tissue pathology in the Plantaris tendon and the surrounding loose connective tissues. Similar Acetylcholine production by tenocytes has previously been shown in painful Achilles and Patellar tendons [16,17]. Plantaris tendon is also reported to be stronger and stiffer than the Achilles tendon [1]. Surgical resection of Plantaris for the management of Mid-AT also has alluded to a possible association and co-existence of pathologies in these two tendons. Masci et al. [18] in their study using ultrasound tissue characterisation showed that Achilles structure improved after resection of Plantaris tendon in Achilles tendinopathy cases.

At present, there are studies to show potential effects of Plantaris tendon morphology in developing Mid-AT, and co-existence of pathologies in both tendons [1,12,15]. Despite this evidence, there is limited reported data on the location of tendon pathology in Achilles and Plantaris tendons when they co-exist. No large cohort investigations describing the pathological characteristics of co-existing PT and Mid-AT have been published. The primary aim of this study is to describe the MRI based pathological characteristics of co-existing PT and Mid-AT among a large cohort of participants diagnosed with Achilles tendinopathy. Identifying normative data for the coexistent pathology of PT and Mid-AT may assist in improving the diagnosis and management of this common and disabling condition.

## 2. Materials and Methods

### 2.1. Study Design and Participants

This was a retrospective observational study involving radiological evaluation of 100 cases of MRI diagnosed Mid-AT. Patients included for analysis were referred with a clinical diagnosis of Achilles tendinopathy and underwent MRI at a large inner-city imaging practice in Melbourne, Australia (Imaging at Olympic Park). Imaging took place between 1 January 2010 and 31 July 2017. Referrals were received from a variety of health practitioners including general practitioners, sports specialist doctors, physiotherapists, and osteopaths. Patients under the age of 18 years and radiologically diagnosed insertional Achilles tendinopathy or acute Achilles rupture were excluded. Ethical approval was obtained from the Human Research Ethics Committee of the La Trobe University, Australia (Project Id: S15-276/S16-276).

### 2.2. Imaging Protocol and Evaluation

All selected MRI scans were performed using the same imaging protocol. A 3T Phillips Ingenia and Achieva scanners were used with a dedicated eight-channel foot and ankle coil. A dedicated coil was used to obtain a high-resolution image with 2.5 mm axial proton density (PD) and PD fat saturated axial images, as well as 2 mm sagittal and coronal PD fat saturated images. All measurements were undertaken using the Intele-viewer radiological software system (Intele-rad medical systems, Version 4-12-1-P45, Montrea, QC, Canada). The selected 100 MRI scans were retrospectively analysed by a musculoskeletal radiologist with over five years of experience in musculoskeletal radiology. The radiologist was blinded to the patient identity and any associated clinical history.

### 2.3. Tendinopathy Definition on MRI

As per the standard imaging definitions of tendinopathy on MRI sequences, tendinopathy was considered to be present whenever there was a signal abnormality and/or increased thickness of the Achilles tendon, evaluated in sagittal and axial images [1]. A tear was differentiated from tendinopathy by the presence of a high-intensity signal (fluid signal on PD and PD fat saturated or STIR images) [19]. The same imaging criteria were used for defining tendinopathy in the Plantaris tendon. MRI scans that showed Paratenon inflammation were excluded from the study due to their lack of sensitivity and specificity to Achilles tendon pathology [20]. The mid-portion of the Achilles was defined as the portion of the tendon located 2–8 cm proximal to its calcaneal insertion [20]. In cases where the Soleus convergence was very distal, and 8 cm of distal free Achilles tendon was not available, the distance between 2 cm from insertion to the convergence of Soleus muscle (distal free tendon) was used to define the midportion of the Achilles tendon in order to achieve uniformity [20]. Since the Achilles tendon inserts over a wide area on the calcaneal bone, for practical reasons, the Achilles insertion point was identified as the most proximal part of the insertion of the Achilles tendon in the sagittal images of the scan.

### 2.4. Outcome Measures

MRI scans were examined for the presence or absence of a Plantaris tendon, and co-existing PT. In the presence of Plantaris, its anatomical characteristics such as the site of tendon insertion were evaluated. Secondly, MRI characteristics for Mid-AT and PT were evaluated. For the Mid-AT, the maximum anteroposterior thickness and the distance from the calcaneal insertion to the epicentre of the abnormality were measured [1]. Similarly, the location of pathology in relation to calcaneal insertion was evaluated for Plantaris tendon, when tendinopathy was noted. When PT co-existed with Mid-AT, the location of the pathology was assessed in relation to the Mid-AT pathology. The anteroposterior thickness of both the Achilles and Plantaris tendons was measured in the axial images, and the distance to the pathology from the calcaneal insertion was measured in the sagittal images [1].

Assessment of MRI images was based on visual inspection. The axial image where each tendon is thickest was identified via visual inspection. Intra-observer reliability of the visual inspection method of thickness measurement was tested by performing blinded repeat measurements on a random sample of 20 tendinopathic Achilles tendons, 10 tendinopathic Plantaris tendons, and 10 normal Plantaris tendons. The cases used for analysis were randomly chosen by one researcher (SK) and evaluated by the same radiologist (AP). To avoid recall bias, the re-evaluation of these cases was done on a day different from the initial evaluation.

### 2.5. Statistical Analysis

The statistical analysis was undertaken using the SPSS version 24 software (IBM Corp. 2016. Armonk, NY, USA). Patient characteristics (e.g., age), and MRI characteristics of tendinopathies were analysed using descriptive statistics and presented as mean ± standard deviation, and range. The prevalence of Plantaris in relation to sex was analysed using the Pearson chi-square test of independence. The relationship between the location of pathology in Mid-AT and PT was evaluated using the coefficient of variation (CV) of their means and Pearson’s correlation coefficient. Assessment of intra-observer reliability of the radiological measurements was conducted using the intra-class correlation coefficient (ICC) method. The significance level was taken at *p* = 0.05.

## 3. Results

The 100 cases of MRI-diagnosed Mid-AT were comprised of 75 male and 25 female patients with a mean age of 43.3 ± 15.8 years (range 19–79 years). Plantaris was identified in 84 cases (84.0%) that include 67 males (89.3% of male patients) and 17 females (68.0% of female patients) with no significant difference in prevalence between the sexes (*p* > 0.05). Of these, 10 cases showed tendinopathy changes in the Plantaris tendon (11.9%), and all were male patients. Intra-observer reliability of radiological measurements of tendons showed high degree of reliability in repeated measures (ICC = 0.99; 95% CI = 0.99–1.00; *p* < 0.001).

A summary of the MRI characteristics (anteroposterior thickness, distance from the calcaneal insertion) of Mid-AT and PT are presented in Table 1. There was no significant association between the Mid-AT thickness (r = −0.03, *p* = 0.79) or location of the pathology (r = 0.06, *p* = 0.53) to the age of the patients.

### 3.1. Location of Mid-AT and PT

**Figure 1:**
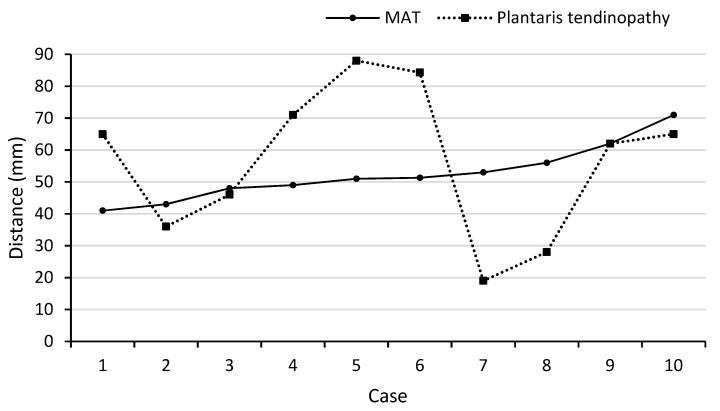
Illustration of the location of Achilles tendon (Mid-AT) and Plantaris tendon (PT) pathology when existing together (n = 10).

**Figure 2:**
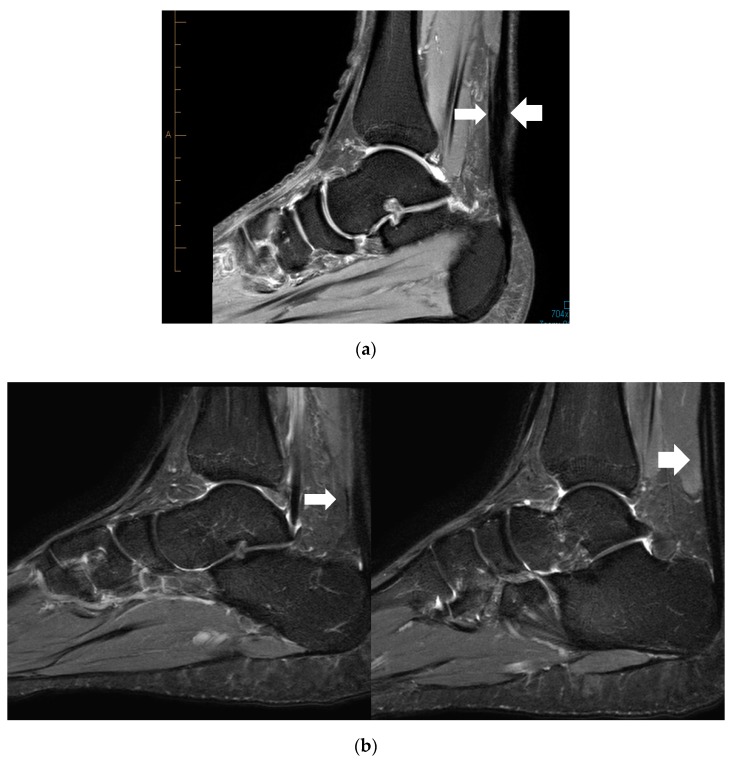
Proton density (PD) sagittal image showing the location of tendinopathic changes in the Achilles (thick arrow) and the Plantaris tendon (thin arrow) at same level (**a**), and at different levels (**b**).

Location of the PT and Mid-AT in relation to their calcaneal insertion is presented in Figure 1 (when these two pathologies are coexisting; n = 10). The mean distance of the two pathologies (PT and Mid-AT) in relation to tendon insertion to calcaneum was similar (Table 1). However, no direct association was observed between the two pathologies and co-located only in one case (Figure 1). The mismatch of Mid-AT and PT pathology locations ranged from −3.7 cm (the Plantaris pathology being more proximal) to 3.4 cm (the Plantaris pathology being more distal). The coefficient of variation (CV = SD/mean × 100) of the distribution of Plantaris pathology (CV = 42%) was greater than the coefficient of variation of Achilles pathology (CV = 17%), which indicates the greater variability in the location of Plantaris pathology than Achilles tendinopathy. The correlation coefficient also revealed a low and non-significant association between the location of two pathologies when they exist together (r = +0.06; *p* = 0.88). Figure 2 demonstrates the different locations of tendinopathic changes in the Achilles tendon (Mid-AT) and Plantaris tendon (PT) using MRI PD sagittal images.

### 3.2. Insertional Anatomy of the Plantaris Tendon (n = 84)

**Figure 3:**
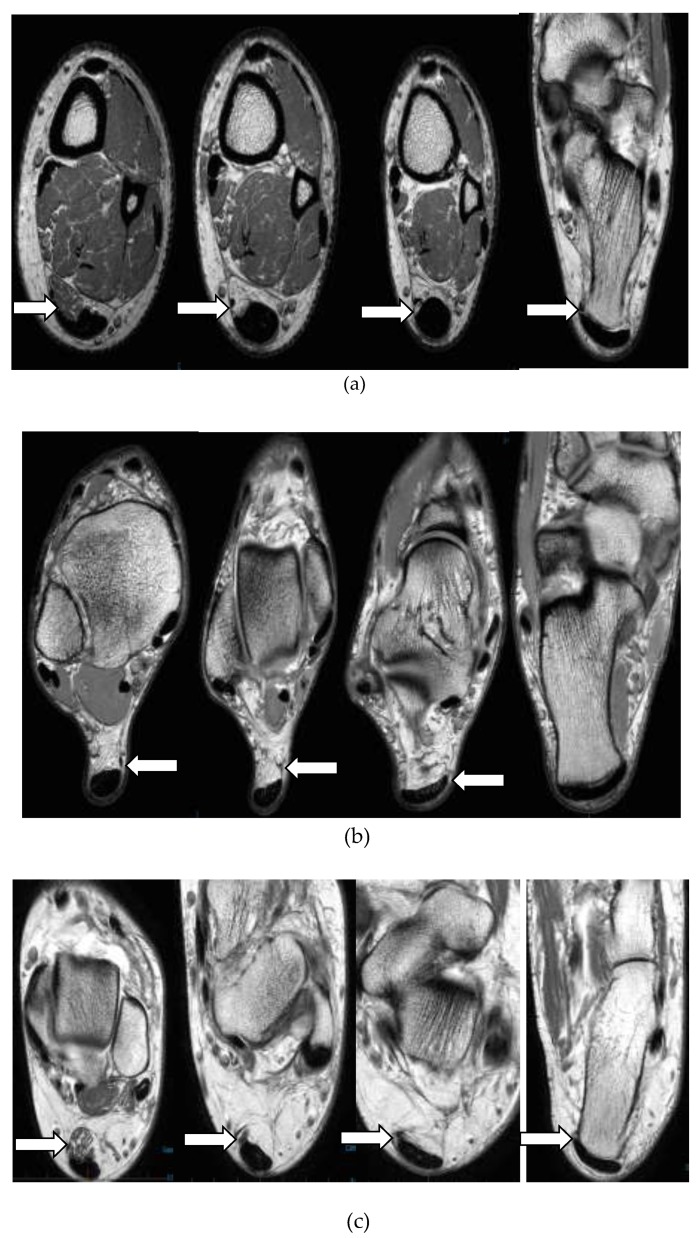
Axial proton density (PD) images showing different insertional characteristics of Plantaris tendon into: calcaneal bone (**a**), Achilles tendon (**b**), both calcaneal bone and Achilles tendon (**c**).

Different Insertional characteristics of Plantaris tendon are illustrated in Figure 3. Plantaris tendon was commonly inserted to the calcaneus (n = 45, 53.6%). In 39 cases (46.4%) tendon was directly inserted to the Achilles tendon. Of these cases, discrete insertions were not identified in seven MRI sequences and observed as inserting to both the Achilles tendon and calcaneal bone (mixed).

## 4. Discussion

To our knowledge, this is the first study to report co-existence of mid-AT and PT based on MRI characteristics in a cohort of patients with Mid-AT. This study showed that one in every 10 patients with radiologically confirmed Mid-AT had co-excitant PT. In relation to the location of pathology in Achilles and Plantaris tendons, no direct association was observed. A considerable variation was observed in the location of the PT, where it was seen both proximal and distal to the location of Mid-AT. There was only one case in our study (of the 10 co-existent pathological tendons) where the pathology in the two tendons was located at the same level. This is in contrast to the findings of an ultrasound and Doppler-guided study by Alfredson [4], which showed co-location and enlargement of the Plantaris pathology in 80% of cases with chronic mid-portion Achilles tendinosis. Further, this differential location of the pathologies in the two tendons refutes the suggestion of compression as a cause of Achilles tendinopathic pain as suggested by Spang et al. [1], who showed pathological Plantaris tendon invaginated into the Achilles tendon. The occurrence of pathology in 10% of cases highlights that plantaris pathology should be looked into during radiological investigations and reported accordingly in suspected or confirmed Achilles tendinopathy cases.

Recent literature has suggested that in cases where Plantaris tendon pathology is co-existent with Achilles tendinopathy, the tenderness is located on the medial side of the Achilles tendon and is more proximal than the mid-portion (in the region of the musculotendinous junction) [5,21]. However, this study did not find such mediolateral location preference of tendinopathic changes in the Achilles tendon in cases with Plantaris tendon pathology. Instead, there was much greater variability (See Figure 1) in the location of the pathology in the Plantaris tendon than the Achilles tendon. Plantaris tendon pathology was located even more distal to the location of the Achilles tendon pathology.

Plantaris tendon was not present in 16 cases (16%). This is consistent with previously published research, where the Plantaris tendon has been found to be absent in about 6–12% [22]. Some of the recent literature, however, has suggested that Plantaris tendon is present in all individuals but was difficult to assess being firmly adhered to the Achilles [23]. We observed a wide variation in the insertion of the Plantaris tendon. The most common insertion was seen in the calcaneum bone followed by in the Achilles tendon. In seven cases, discrete insertion of Plantaris was not identified on MRI evaluation. However, cadaveric studies have described detailed insertional characteristics of Plantaris and include: calcaneum bone, medial part of Achilles tendon, dorsal part of the Achilles tendon at its insertion, deep and superficial fascia of the leg, fibro-fatty tissue around the Achilles tendon and plantar aponeurosis [5,12,13,24,25]. Studies by Olewnik et al. [12,13] have described six insertion patterns based on cadaveric dissections. These insertions were primarily on different anatomical areas of the calcaneus and the Achilles’ tendon. In a minority of cases, Plantaris tendon was identified to insert into the fascial structure adjoining the Achilles’ tendon (3.4%) and to the flexor retinaculum at the ankle (5.2%). In another similar cadaveric study of Plantaris insertion, nine different Plantaris tendon insertional patterns were identified [5]. These insertions also described different anatomical locations on the calcaneus, Achilles’ tendon and the deep fascia. Based on these anatomical/cadaveric studies, it is apparent that MRI sequences have limitations in defining the insertional patterns in detail. Further, prior to our study, we have not found studies describing insertional characteristics of Plantaris tendon on MRI scans in the scientific literature. Further radiological studies are needed in the future to understand the ability of MRI-based data to provide descriptive information on Plantaris insertional characteristics. Studies by Olewnik et al. [12,13] have suggested that insertion patterns of Plantaris tendon and its course in the leg can play a role in Achilles tendinopathy. These anatomical variations were found on cadaveric studies and this is an area of further prospective clinical research to potentially define such a causal relationship.

In this study, only male subjects showed the involvement of the Plantaris in association with Achilles tendinopathy. It is not clear from our observations if this condition only occurs in males or is skewed because of the high number of male subjects in the sample and/or if it reflects particular sporting participation. This is an area requiring further investigation. It is difficult to differentiate Plantaris tendinopathy from Achilles tendinopathy clinically, and it is yet to be ascertained if image guided local Anaesthetic injections can differentiate the pain generator in co-existent pathologies in plantaris and Achilles tendons. Prospective studies in the future are required to establish rehabilitation protocols in cases of co-existent pathologies.

### 4.1. Strengths of the Study

This study utilised high-resolution 3T MRI scanning of 100 participants. Intra-observer reliability for identifying the pathological site in the tendon was tested and found to be strong. This study has confirmed previous data on the prevalence of Plantaris and added imaging evidence to the knowledge of potential insertional patterns of the Plantaris tendon. 

### 4.2. Limitations of the Study

In this study, we did not co-relate the image findings to the clinical findings, so cannot comment on the site and cause of pain in Achilles tendinopathy. MRI may be less sensitive to mild intra-tendinous pathology than Ultrasound scan. Since the cases included in the study already had a clinical diagnosis of Achilles tendinopathy, it is possible that many cases of Achilles tendinopathy were treated without imaging and therefore could not be included in this research. If all those cases were also included, it is possible that a different prevalence of Plantaris involvement or pattern of tendinopathy could have been identified. Patients were referred from external clinicians; therefore, details of sporting activity, clinical presentation, and previous treatments were not available to the researchers to derive any conclusions. The image sequences used in the study evaluated only the lower third of the leg. Potentially subjects that had a high insertion of Plantaris into the Achilles tendon could have been missed and categorised as an absent Plantaris.

## 5. Conclusions

Tendinopathic changes in the Plantaris tendon are present in 10% of cases of mid-AT. These changes may play a role in causing Achilles tendon pain and are possibly contributing to cases where tendons are resistant to the standard treatment of Achilles tendinopathy. Therefore, clinical evaluation of Achilles tendon pain needs careful consideration into the possible co-existence of Plantaris pathology, especially in the absence of radiological evaluation. The difference observed in the location of PT and Mid-AT suggest possible isolated pathologies and differentials for Achilles tendon pain. Further research is required to determine the significance of different patterns of Plantaris insertion and whether these play a role in the development of Achilles tendon symptoms or Achilles tendinopathy.

## Figures and Tables

**Table 1:** MRI characteristics (anteroposterior thickness and distance from the calcaneal insertion) of Mid-AT and PT.

Measurement	Statistical Parameter	Mid-AT (n = 100)	Mid-AT When Coexist with PT (n = 10)	PT When Coexist with Mid-AT (n = 10)
Antero-posterior thickness ^a^ (mm)	Mean ± SD	10.0 ± 3.0	11.5 ± 2.5	4.1 ± 0.1
	Range	5.5–23.0	6.7–14.3	2.5–5.6
	Median	9.8	12.2	4.2
Distance from the calcaneal insertion ^b^ (mm)	Mean ± SD	44.1 ± 13.2	52.5 ± 9.0	56.1 ± 23.7
	Range	20.0–71.3	40.6–71.3	19.5–87.6
	Median	43.9	51.0	63.3

^a^ Measured in the axial plane on PD images; ^b^ Measured in the sagittal plane on PD images.
